# The Adequate Corpus Luteum: miR-96 Promotes Luteal Cell Survival and Progesterone Production

**DOI:** 10.1210/jc.2017-00259

**Published:** 2017-03-20

**Authors:** Bushra T. Mohammed, Sadanand D. Sontakke, Jason Ioannidis, W. Colin Duncan, F. Xavier Donadeu

**Affiliations:** 1 The Roslin Institute and Royal (Dick) School of Veterinary Studies, University of Edinburgh, Easter Bush, Midlothian EH25 9RG, United Kingdom; 2 The Queen's Medical Research Institute, MRC Centre for Reproductive Health, Edinburgh EH16 4TJ, United Kingdom

## Abstract

**Context:**

Inadequate progesterone production from the corpus luteum is associated with pregnancy loss. Data available in model species suggest important roles of microRNAs (miRNAs) in luteal development and maintenance.

**Objective:**

To comprehensively investigate the involvement of miRNAs during the ovarian follicle-luteal transition.

**Design:**

The effects of specific miRNAs on survival and steroid production by human luteinized granulosa cells (hLGCs) were tested using specific miRNA inhibitors. Candidate miRNAs were identified through microarray analyses of follicular and luteal tissues in a bovine model.

**Setting:**

An academic institution in the United Kingdom associated with a teaching hospital. hLGCs were obtained by standard transvaginal follicular-fluid aspiration from 35 women undergoing assisted conception.

**Intervention(s):**

Inhibition of candidate miRNAs *in vitro*.

**Main outcome measure(s):**

Levels of miRNAs, mRNAs, FOXO1 protein, apoptosis, and steroids were measured in tissues and/or cultured cells.

**Results:**

Two specific miRNA clusters, miR-183-96-182 and miR-212-132, were dramatically increased in luteal relative to follicular tissues. miR-96 and miR-132 were the most upregulated miRNAs within each cluster. Database analyses identified FOXO1 as a putative target of both these miRNAs. In cultured hLGCs, inhibition of miR-96 increased apoptosis and FOXO1 protein levels, and decreased progesterone production. These effects were prevented by small interfering RNA-mediated downregulation of FOXO1. In bovine luteal cells, miR-96 inhibition also led to increases in apoptosis and FOXO1 protein levels.

**Conclusions:**

miR-96 targets FOXO1 to regulate luteal development through effects on cell survival and steroid production. The miR-183-96-182 cluster could provide a novel target for the manipulation of luteal function.

## Teaser

The involvement of miRNAs in the follicle-luteal transition was investigated. miR-96 was found to promote luteal cell survival and progesterone synthesis by luteinized granulosa cells.

In monovular species, such as humans, ovulation involves rupture of the wall of a mature follicle and release of the contained oocyte for fertilization. After ovulation, the follicular remnants undergo profound remodeling, resulting in formation of a highly vascular, highly steroidogenic corpus luteum (CL), with a critical role in establishment and maintenance of pregnancy. Luteal development involves fine-tuned changes in proliferation, survival, migration, and differentiation, simultaneously affecting many cell types (1). Particularly critical is the differentiation of estrogen-producing follicular cells into luteal cells with the ability to produce high levels of progesterone. The importance of this is reflected in the association of luteal insufficiency and/or suboptimal progesterone levels with pregnancy failure in species including cattle, sheep, and horses (2–4). Although a similar association has been proposed in humans (5), controversy exists about whether the CL is actually a primary cause of infertility in women (6). A much-needed understanding of molecular regulation of luteal development in humans would provide clarification and assist in identifying therapeutic targets for fertility manipulation.

miRNAs are ubiquitously involved in posttranscriptional gene regulation during tissue development and differentiation. Global microRNA (miRNA) profiles during follicular development have been reported in cattle, sheep, and mice (7–9), and numerous miRNAs were shown to regulate follicular proliferation, survival, and steroidogenesis (10–12). In addition, a substantial number of miRNAs change in expression during the follicle-luteal transition and luteal maturation (7, 9, 13, 14); however, although specific roles have been demonstrated for some of these miRNAs in rodents, including in luteal angiogenesis [miR-17-5p and let-7b (15)], survival [miR-21 (16)], and luteinizing hormone/choriogonadotropin receptor downregulation [miR-136-3p and miR-122 (17, 18)], very limited information exists on their involvement in other species, particularly humans (14).

A greater understanding of the roles of miRNAs in normal luteal development—in particular, the follicle-luteal transition—could provide important insight into human reproductive health. Considering this, we performed analysis both in cells from human patients and in tissues collected from cattle. Being a monovular species, cattle provide a convenient model to study human ovarian physiology, allowing study of clinically relevant follicular and luteal tissues difficult to access in women. Our studies identified an miRNA cluster that is highly expressed in the CL and plays a role in promoting luteal cell survival and steroidogenesis, providing a potential target for future interventions in human reproductive health.

## Methods

### Tissue collection

Human luteinized granulosa cells (hLGCs) were obtained (19) from 35 donors undergoing assisted conception at the Simpson Centre for Reproductive Health, Royal Infirmary of Edinburgh, United Kingdom. Ethical approval was given by the regional medical research ethics committee (2005/R/RM/11 and SR431), all women gave informed consent, and cells were analyzed anonymously. Cells were centrifuged through Ficoll Paque Plus solution (1.077 g/cm^3^; GE Healthcare, Buckinghamshire, UK), then the middle layer was collected and washed before culturing. Cell viability was ∼70%.

Bovine tissues were collected at an abattoir. Ovarian pairs containing a visible CL were used to collect individual follicles >10 mm in diameter (8). Follicular walls and follicular fluid were snap frozen in liquid nitrogen and frozen at −80°C, respectively. CLs corresponding to days 1 to 4 of an estrous cycle (20) were collected and snap frozen. Bovine granulosa cells were obtained from follicles 4 to 8 mm in diameter (8, 21). Luteal cells (*i.e.*, steroidogenic and other cells, including fibroblasts, endothelial, and immune cells) were isolated using a modification of published protocols (22). In brief, the CL was minced and digested twice in collagenase-II and bovine serum albumin (both from Sigma-Aldrich, Irvine, UK) at 37°C for 45 minutes. Supernatants were filtered (100-µm filters) and then incubated with DNase-I (Sigma-Aldrich) for 10 minutes at 37°C. After digestion, cells were filtered again (70-μm filters), washed, and incubated with red cell blood lysis buffer (8.3 g/L NH_4_Cl in 0.01 M Tris-HCl; pH, 7.5; Sigma-Aldrich) for 1 minute. Cell viability was >80%.

### Cell culture

hLGCs and bovine luteal cells were cultured in 24- or 12-well plates (100,000 and 500,000 cells per well; Thermo Fisher Scientific, Perth, UK) in Dulbecco’s modified Eagle medium/F-12 (Thermo Fisher Scientific) containing 2.5 mM l-glutamine; 15 mM HEPES; 1% penicillin-streptomycin; fetal bovine serum (FBS; 10% volume-to-volume ratio for the first 24 hours, and 2% thereafter); insulin (5 μg/mL), transferrin (5 μg/mL), and sodium selenite (5 ng/mL); and amphotericin b (2.5 μg/mL; all from Sigma-Aldrich) in a humidified atmosphere at 37°C with 5% CO_2_. Twenty-four hours later, cells were transfected with locked nucleic acid (LNA) anti-miRNAs (50 nM; Exiqon, Vedbaek, Denmark), two human FOXO1 small interfering RNAs (siRNAs; catalog no. 106654 and 106653; 100 nM each; Thermo Fisher Scientific) and/or a scrambled oligonucleotide (50 or 100 nM; AllStars Negative Control; Qiagen, Manchester, UK) using Hiperfect reagent (Qiagen). One or 2 days later, they were collected for RNA, protein, or Caspase 3/7 activity analyses while culture media were frozen. In some instances, after 6 days in culture, hLGCs were treated with human chorionic gonadotropin (hCG; 100 ng/mL; Merck Serono, Feltham, UK) for 4 days, after which RNA was collected. Progesterone and estradiol levels in culture media were quantified using Coat-A-Count radioimmunoassay (Siemens Healthcare Diagnostics, Erlangen, Germany) and DIAsource E2-RIA-CT (DIAsource ImmunoAssays, Ottignies-Louvain-la-Neuve, Belgium) kits, respectively. Sensitivities and coefficients of variance were 0.01 ng/mL and 1 pg/mL, and 4.3% and 2.6%, respectively, for each assay.

Bovine granulosa cells were cultured as described (21) and, 24 hours later, were either left untreated or treated with forskolin (10 μM), bovine insulin (1 mg/mL), and FBS (1% volume-to-volume ratio; all from Sigma-Aldrich) for up to 4 days to induce luteinization.

### RNA extraction

Total RNA was extracted from snap-frozen tissues using the miRNeasy Mini kit (Qiagen) after homogenization with ceramic beads using FastPrep FP120 cell disruptor (MP Biomedicals, Luton Bedford, UK). RNA was analyzed using a NanoDrop-1000 spectrophotometer (NanoDrop Technologies, Wilmington, DE) and the Agilent 2100 Bioanalyzer (Agilent Technologies, Santa Clara, CA). RNA from cultured cells was isolated using TRIzol Reagent (Thermo Fisher Scientific) and concentrations were determined using the Quant-iTRiboGreen RNA kit (Thermo Fisher Scientific).

### Microarray analyses

Bovine samples from six large (12- to 17-mm diameter), steroidogenically active follicles [classified based on *CYP19A1* and estradiol levels (8)] and six early CLs were analyzed with the miRCURY LNA microRNA array sixth generation (contained 1488 capture probes targeting all miRNAs for human, mouse, or rat in miRBase 16.0; Exiqon Services, Denmark), as described in detail (8). Differences in miRNA expression were determined using Student *t* test with Benjamini and Hochberg false discovery rate adjustment. Raw microarray data were deposited in NCBI’s National Center for Biotechnology Information Gene Expression Omnibus repository (gene accession no. GSE54692).

### Quantitative reverse transcription-polymerase chain reaction

Individual miRNAs were analyzed using miScript II RT and miScript SYBR Green PCR kits, and miScript Primer Assays (Qiagen). mRNA levels were quantified on the same cDNA using species-specific primers (Supplemental Table 1) and the SensiFAST SYBR Lo-ROX Kit (Bioline, London, UK). The MX3005P QPCR system (Stratagene, La Jolla, CA) was used. Relative transcript abundance was obtained using MX3005P software by extrapolating cycle threshold values from a standard curve prepared from a sample pool. Endogenous *RnU6-2* was used for normalization of miRNA, and 18S or *GAPDH* were used to normalize mRNA data.

### *In situ* hybridization

*In situ* hybridization of frozen ovarian tissues was performed using a modified protocol (8) with double digoxigenin-labeled LNA probes (Exiqon) against bta-miR-132 (80 nM), *RnU6-2* (3 nM), or a scrambled RNA sequence (40 nM). Independent analyses were performed on three different sections.

### Western blotting

Total protein was obtained by adding buffer (4% sodium dodecyl sulfate, 20% glycerol, 10% 2-mercaptoethanol, 0.004% bromophenol blue and 0.125 M Tris HCl; pH, 6.8; Sigma-Aldrich) to cultured cells at 60°C and then scraping off. Samples were boiled for 5 min and electrophoresed in a 12% sodium dodecyl sulfate–polyacrylamide gel electrophoresis gel with color-plus prestained marker (BioRAD, Watford, UK) in Mini Trans-Blot Cell (BioRAD) at 150 V for 90 minutes. Gels were transferred to a 0.2 µM nitrocellulose membrane (GE Healthcare) using Trans-Blot SD Semi-Dry Transfer Cell (BioRAD) at 15 V for 60 minutes. After blocking, the blot was incubated with anti-FOXO1 (1:500; catalog no. 2880; Cell Signaling, Danvers, MA) or anti-*β*-tubulin (1:1000; catalog no. 2146; Cell Signaling) overnight at 4°C, followed by washing and incubation with IRDye 680RD donkey anti-rabbit IgG (1:10,000; catalog no. 926-68073; LI-COR Biosciences, Cambridge, UK) for 1 hour and visualization with a LI-COR Odyssey IR imaging scanner. Signal intensities were quantified using Image Studio Lite 5.0 (LI-COR).

### Apoptosis assays

Apoptosis was measured in triplicate in 96-well plates (2 × 10^4^ cells per well) using Caspase-Glo 3/7 assays (Promega, Madison, WI) and a Synergy microplate reader (BioTek, Winooski, VT). In addition, cells grown on coverslips were stained with Annexin-V-Fluos staining kit (Roche, Burgess Hill, UK) and visualized using a Leica DMLB fluorescence microscope (Leica Microsystems, Buffalo Grove, IL).

### Statistical analyses

Data were analyzed using the GLM procedure by one-way or two-way analysis of variance followed by Tukey pairwise comparison tests or, whenever only two experimental groups were compared, Student *t* tests. In all cases, statistical significance was considered at *P* < 0.05.

## Results

### miR-183-96-182 and miR-212-132 clusters are highly upregulated during the follicular-luteal transition

**Figure 1.**
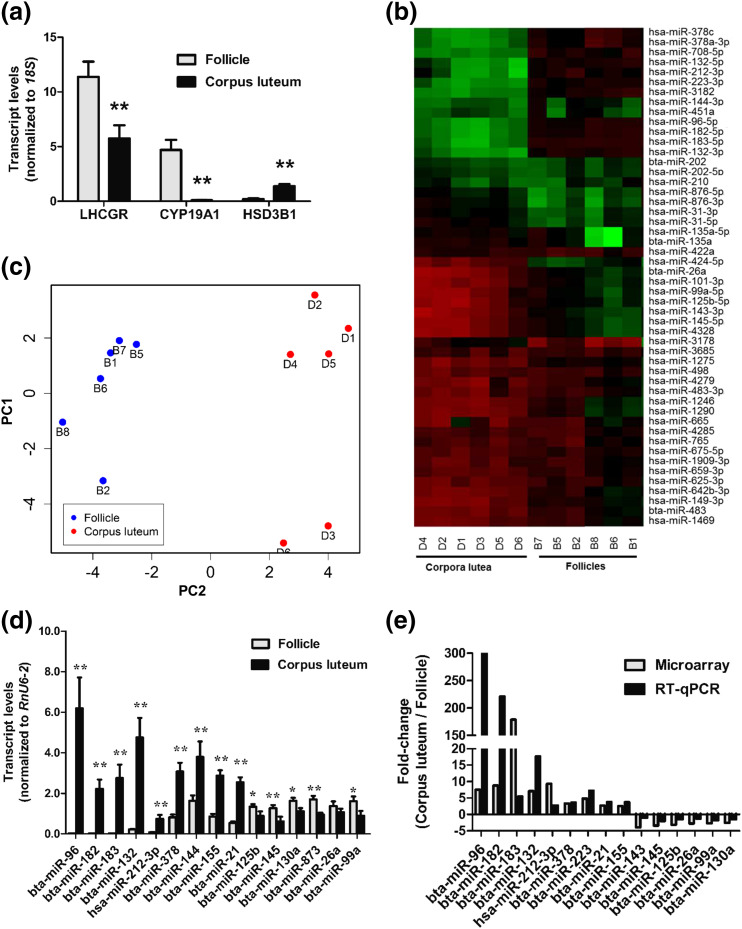
(a) Relative mean [± standard error (SE)] transcript levels of *LHCGR*, *CYP19A1*, and *HSD3B1* in bovine ovulatory-size follicle (12 to 17 mm; n = 6) and early CL (n = 6) samples used for microarray analyses. (b, c) Heat map representation (b) and principal component analyses (PCA) plot (c) of top 50 miRNA probes with highest standard deviation in bovine ovulatory-size follicles and early CLs. Each row in the heat map represents an miRNA and each column represents a sample. The color scale illustrates the relative expression level of miRNAs. Red represents an expression level below the reference channel and green represents expression higher than the reference. For PCA plot analysis, the normalized log ratio values were used. The features were shifted to be zero centered (*i.e.*, the mean value across samples was shifted to 0) and scaled to have unit variance (*i.e.*, the variance across samples was scaled to 1 before the analysis). Raw microarray data were deposited in the National Center for Biotechnology Information Gene Expression Omnibus repository (GSE54692). (d) Levels (mean ± SE), obtained by qPCR, of top transcripts identified by microarray as up- or downregulated in early CL relative to ovulatory-size follicles. (e) Comparative mean fold changes in miRNA expression between bovine early CL and ovulatory-size follicles obtained by microarray and qPCR. P values are for the differences between two means, determined by *t* test. *P < 0.05; **P < 0.01.

To identify miRNAs potentially involved in the follicular-luteal transition in the monovular ovary, we collected bovine large antral follicles and early-cycle CLs. Expression profiles of selected genes were consistent with those naturally encompassing the follicle-luteal transition [Fig. 1(a)]. Upon microarray analyses, a total of 545 probes yielded hybridization intensities above background across all samples, corresponding to 523 unique miRNAs, including 191 sequences registered as bovine in miRBase 18. Results of comparative analyses are shown in Figure 1(b) and 1(c) and Supplemental Table 2.

A total of 11 and 22 unique miRNAs were up- and downregulated, respectively (≥2.5-fold; false discovery rate, <0.01) in CLs relative to large antral follicles (Table 1). The top four differentially expressed sequences corresponded to the homologs of human miR-183-5p, miR-96-5p, miR-182-5p, and miR-132-3p, and were all upregulated in the CL [Fig. 1(d) and 1(e)]. These sequences derive from two different miRNA clusters: miR-183-96-182 and miR-212-132. Only one of the -3p homologs in the miR-183-96-182 cluster, miR-183, was also detected by microarray, and was slightly upregulated in the CL (*i.e.*, 1.15-fold; Supplemental Table 2). In addition, the homologs of human miR-132-5p and miR-212-3p (none registered as a bovine sequence in miRBase 18), but not miR-212-5p, were also detected and were upregulated in the CL [Table 1; Fig. 1(d) and 1(e)]. Because bta-miR-96 and bta-miR-132 were the top upregulated miRNAs within each cluster [Fig. 1(d)] and, based on cycle-threshold values, were also the most abundant in ovarian tissues, our subsequent analyses focused on these two miRNAs (hereafter referred to as miR-96 and miR-132, for simplicity).

**Table 1.** miRNAs Differentially Expressed (≥2.5-Fold) Between Early CLs and Preovulatory-Size Follicles in Cow

**miRNA^*a*^,^*b*^,^*c*^**	**Fold Change**
Upregulated in CL	
hsa-miR-182-5p/bta-miR-182	8.80
hsa-miR-96-5p/bta-miR-96	7.55
hsa-miR-132-3p/bta-miR-132	7.11
hsa-miR-183-5p/bta-miR-183	5.42
hsa-miR-223-3p/bta-miR-223	4.79
hsa-miR-378a-3p/hsa-miR-378c/hsa-miR-378d/bta-miR-378	3.35
hsa-miR-3182	3.30
hsa-miR-132-5p	3.20
hsa-miR-708-5p/bta-miR-708	3.11
hsa-miR-212-3p	2.71
bta-miR-21	2.67
Downregulated in CL	
hsa-miR-1290	5.24
hsa-miR-1246	4.42
hsa-miR-143-3p/bta-miR-143	3.93
hsa-miR-642b-3p	3.85
hsa-miR-4328	3.63
hsa-miR-145-5p/bta-miR-145	3.40
hsa-miR-125b-5p/bta-miR-125b	3.12
hsa-miR-195-5p/bta-miR-195	3.03
hsa-miR-149-3p	2.98
hsa-miR-424-5p	2.96
hsa-miR-574-5p	2.92
hsa-miR-10b-5p/bta-miR-10b	2.83
bta-miR-26a	2.76
hsa-miR-99a-5p/bta-miR-99a	2.73
hsa-miR-491-3p	2.67
bta-let-7b	2.65
hsa-miR-100-5p/bta-miR-100	2.60
hsa-miR-130a-3p/bta-miR-130a	2.59
hsa-miR-101-3p/bta-miR-101	2.52
hsa-miR-125a-5p/bta-miR-125a	2.52
hsa-miR-32-3p	2.51
hsa-miR-30c-5p/bta-miR-30c	2.51

^a^ miRNA nomenclature according to miRBase 21.

^b^ n = 6 animals per tissue type.

^c^ *P* < 0.01 (Benjamini and Hochberg adjusted) in all cases.

**Figure 2.**
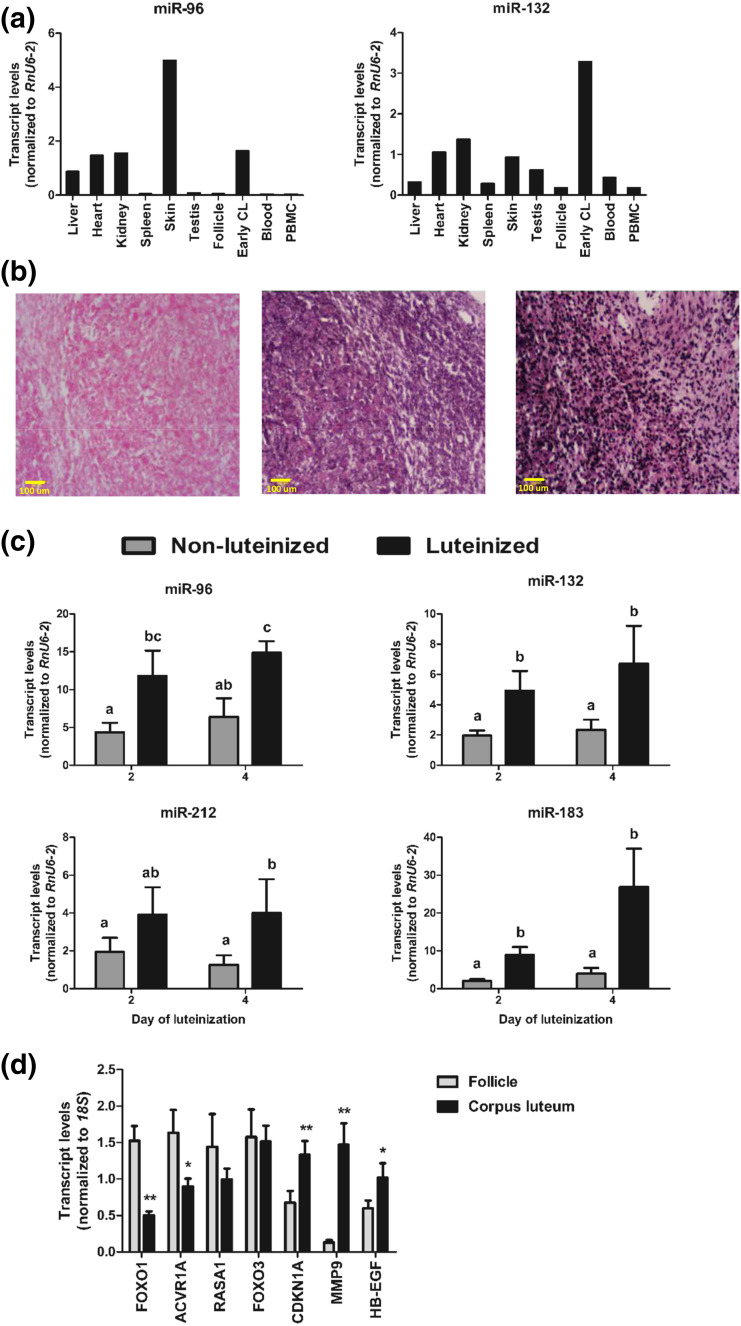
(a) Relative abundance of miR-96 and miR-132 across several bovine tissues. For each tissue, RNA samples from three to five animals were pooled and analyzed by qPCR. (b) Localization of miR-132 within the bovine CL by *in situ* hybridization. Frozen ovarian sections were hybridized with digoxigenin-labeled LNA probe against scrambled RNA (negative control, left panel), bta-miR-132 (middle panel), and RnU6 snRNA (positive control, right panel). Scale = 100 μm; ×20 magnification. (c) Changes in relative miRNA levels (mean ± SE) during *in vitro* luteinization of bovine granulosa cells. Granulosa cells were cultured for 4 days with or without media containing forskolin, insulin, and FBS to induce luteinization (n = 5 experiments). Data are shown relative to expression values on day 0. Group means with different letters (a, b, c) are different (*P* < 0.05). (d) Relative mean (±SE) transcript levels, quantified by qPCR, of predicted common targets of miR-96 and miR-132 in bovine ovulatory-size follicles (9 to 17 mm; n = 6) and early CL (n = 6). *P < 0.05 for mean differences within each gene. PBMC, peripheral blood mononuclear cell.

Quantitative polymerase chain reaction (qPCR) screening across bovine tissues revealed neither miR-96 nor miR-132 was restricted to the ovary [Fig. 2(a)]. Nevertheless, miR-132 was expressed at highest levels in CL, although *in situ* hybridization showed that within the CL, this miRNA was broadly distributed and not restricted to any particular cell type [Fig. 2(b)].

Next, to ascertain whether the increase in miR-96 and miR-132 during luteinization involves granulosa-derived cells, the major source of luteal progesterone, we induced bovine granulosa cells to luteinize in culture (7) and showed that, indeed, this was associated with a distinct increase in the expression of miR-96, miR-132, and other miRNAs from the same genomic clusters [Fig. 2(c)].

### FOXO1 is a putative target of miR-96 and miR-132 during the follicle-luteal transition

To investigate the roles of miR-96 and miR-132, we first used TargetScan 7.1 (Whitehead Institute for Biomedical Research, Cambridge, MA) and miRTarBase 6.0 (ISBLab, National Chiao Tung University, Hsinchu, Taiwan) to obtain lists of computationally predicted targets in humans and bovines, and experimentally validated targets (available from humans and rodents), respectively. To identify high-confidence targets, we selected a subset of genes that (1) were predicted targets of both miRNAs, (2) contained conserved target sites, and (3) were known to be involved in luteal development (Ovarian Kaleidoscope Database; http://okdb.appliedbioinfo.net/), and we then determined their relative expression in ovarian tissues [Fig. 2(d)]. In this way, the transcription factor, *FOXO1*, a critical regulator of cell survival and metabolism (23), was identified as high-confidence target based on its clearly decreased expression in CL relative to follicles. The levels of other genes analyzed were only slightly lower (*i.e.*, *ACVR1A*), not different (*i.e.*, *RASA1* and *FOXO3*) or higher (*i.e.*, *CDKN1A*, *MMP9*, and *HB-EGF*) in CL than in follicles, indicating they may not naturally mediate the effects of those miRNAs during the follicle-luteal transition.

### miR-96 has an antiapoptotic effect in hLGCs mediated by FOXO1

**Figure 3.**
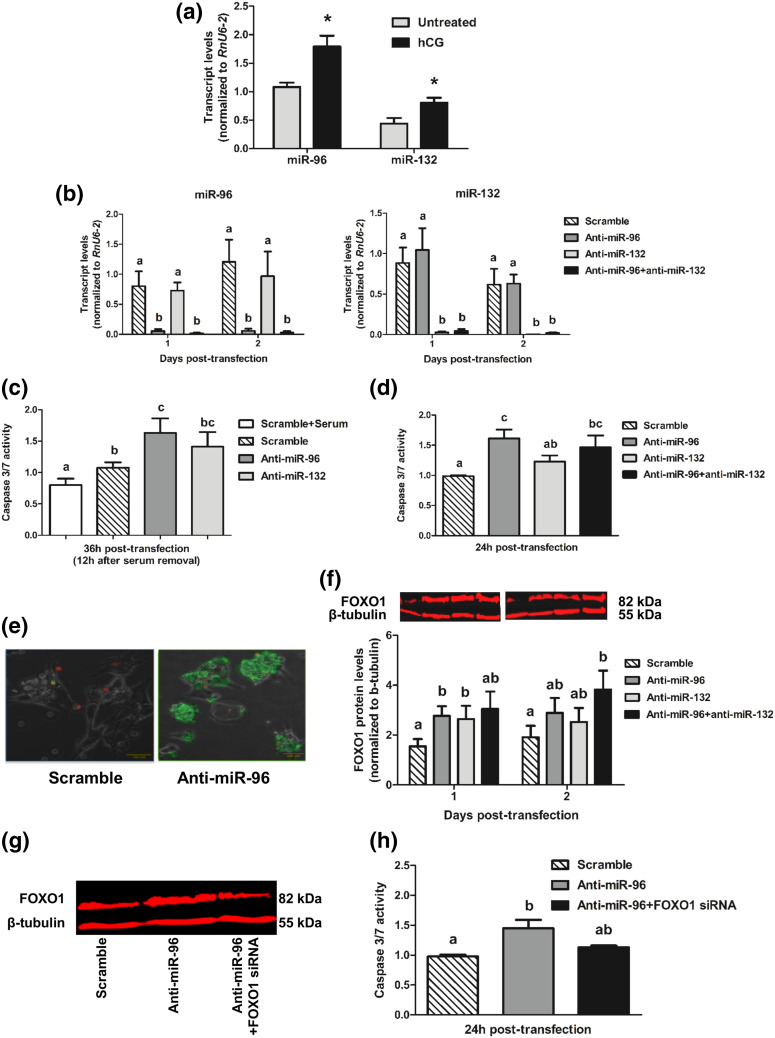
(a) Relative levels of miR-96 and miR-132 in hLGCs treated with hCG (100 ng/mL) for 4 days or left untreated. *P < 0.05 for difference between two means. (b) Relative levels of miRNAs following transfection of hLGCs with the indicated oligonucleotides. (c, d) Caspase 3/7 activity in transfected hLGCs (c) with or (d) without previous serum removal for 12 hours. (e) Representative pictures (scale = 100 μm; ×20 magnification) showing annexin V staining (green; propidium iodide staining is shown in red) of hLGCs 24 hours after transfection with the indicated oligonucleotides. (f) Relative levels of FOXO1 protein after transfection of hLGCs with the indicated oligonucleotides. A representative western blot is shown. Mean FOXO1 values were compared across groups within each day. (g) Representative FOXO1 and *β*-tubulin western blots of hLGCs transfected with the indicated oligonucleotides for 1 day. (h) Caspase 3/7 activity after transfection of hLGCs with the indicated oligonucleotides. In all experiments, oligonucleotides were transfected at the following concentrations: 50 or 100 nM of scramble oligonucleotide (negative control), 50 nM anti-miR-96, 50 nM anti-miRNA-132, 25 nM both anti-miR-96 plus anti-miR-132, and 100 nM FOXO siRNA. Values shown for each time point were normalized in all cases to the corresponding value at the time of transfection (day 0 or hour 0). Data are shown as mean ± SE (n = 3 to 6 experiments). For comparisons involving more than two means, different letters (a, b, c) are used to indicate significance (*P* < 0.05).

To investigate the involvement of these miRNAs in the human ovary, we first determined changes in miRNA expression in hLGCs (corresponding to an early stage of luteinization) that had been treated with hCG to induce further differentiation in culture (24). Results showed that, as in bovine cells [Fig. 2(c)], the two miRNAs were upregulated in response to a luteinization stimulus in human cells [Fig. 3(a)].

Next, we investigated whether, as suggested by the results of our miRNA target analyses, these two miRNAs may regulate luteal cell survival. We transfected hLGCs with anti-miRNAs [Fig. 3(b)] and determined the effects on apoptotic responses to serum removal. We found that anti-miR-96, but not anti-miR-132, led to a significant mean increase (1.6-fold) in the Caspase 3/7 activation response to serum starvation [Fig. 3(c)]. Interestingly, similar Caspase 3/7 responses were obtained even in nonstressed cells maintained in serum; in addition, under those conditions, simultaneous inhibition of both miRNAs produced a Caspase 3/7 response similar to that induced by inhibition of miR-96 only [Fig. 3(d)]. The proapoptotic effect of miR-96 inhibition was confirmed by annexin V staining [Fig. 3(e)].

We then determined whether the effects of miR-96 could be mediated by its putative target, FOXO1. Indeed, inhibition of miR-96 induced a robust mean increase (1.8-fold) in FOXO1 protein 1 day after transfection [Fig. 3(f)], with a slightly smaller (1.7-fold) although significant increase induced also by miR-132 inhibition. Again, simultaneous inhibition of the two miRNAs did not have a synergistic effect on FOXO1 levels. To confirm a causal involvement of FOXO1 in the observed apoptotic response to anti-miR-96, we transfected cells simultaneously with anti-miR-96 and FOXO1 siRNA. We showed that this effectively prevented both an increase in FOXO1 protein [Fig. 3(g)] and the activation of Caspase 3/7 [Fig. 3(h)] in response to anti-miR-96, thus indicating that miR-96 promotes hLGC survival by targeting FOXO1.

### miR-96 promotes progesterone production by hLGCs through targeting FOXO1

**Figure 4.**
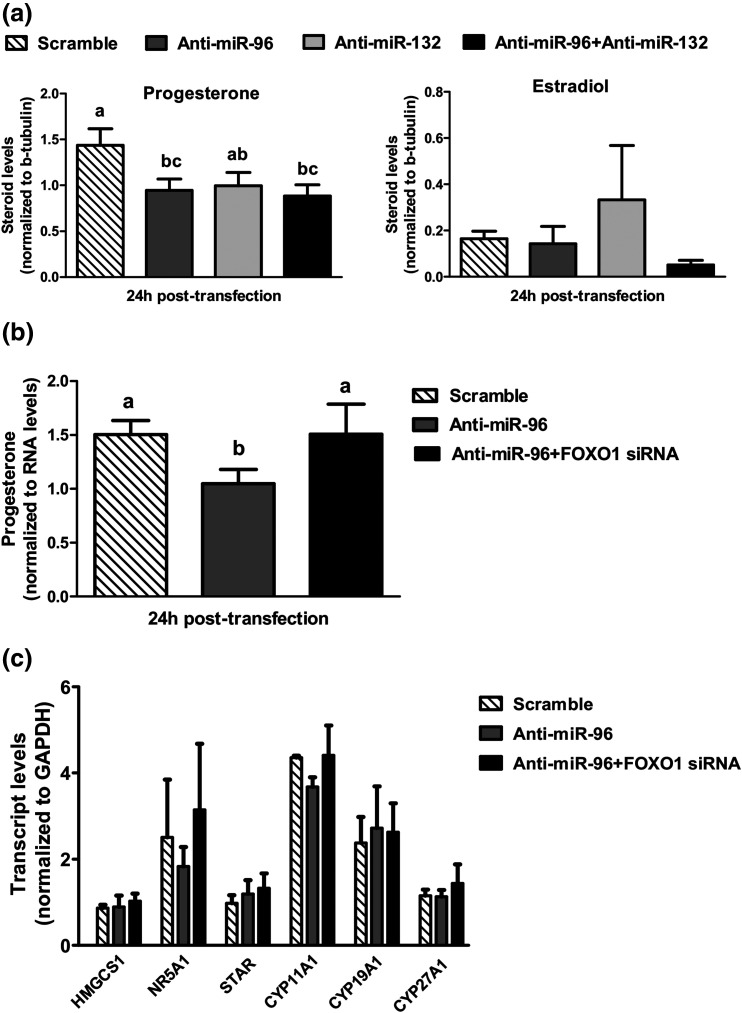
Levels of progesterone (a, b) and estradiol (a) in spent culture media 1 day after transfection of hLGCs with the indicated oligonucleotides (n = 6 experiments). (c) Transcript levels of different genes involved in cholesterol and steroid production, quantified by qPCR in hLGC samples collected 1 day after transfection with the indicated oligonucleotides (n = 3 experiments). Mean (±SE) values are shown and were normalized to values on the day of transfection (day 0). Group means with different letters (a, b, c) are significantly different (*P* < 0.05).

Given the reported involvement of miRNAs in steroidogenesis (10, 11), we investigated the short-term effects of miR-96 and miR-132 on progesterone and estradiol production, by analyzing spent culture media of hLGCs transfected with anti-miR-96 and/or anti-miR-132 [Fig. 4(a)]. All treatments resulted in a decrease in mean progesterone levels at 24 hours; however, this was significant only in response to anti-miR-96, alone or in combination with anti-miR-132 (>1.6-fold; *P* < 0.01). Moreover, the effects of anti-miRs on progesterone were transient; differences were no longer detected 48 hours after transfection (*P* > 0.1; not shown). In contrast, significant changes in estradiol levels were not detected (*P* > 0.1) in response to transfection with anti-miRNAs.

We then determined whether the observed stimulatory effects of miR-96 on progesterone may involve repression of FOXO1. This was, indeed, the case, because transfection with FOXO1 siRNA prevented the temporary reduction in progesterone levels by anti-miR-96 [Fig. 4(b)], indicating that, in hLGCs, an inhibitory effect of FOXO1 on progesterone synthesis is relieved by an increase in miR-96 upon luteinization.

To investigate the mechanisms behind the observed effects of miR-96 and FOXO1 on steroid levels, we quantified the expression of several genes involved along the cholesterol and steroid synthesis pathways [Fig. 4(c)], the transcript levels of which were previously shown to be regulated by FOXO1 in rodent granulosa cells (25). We did not detect significant differences in the levels of any of the transcripts analyzed in response to inhibition of miR-96 in the absence or presence of FOXO siRNA, indicating that distinct molecular mechanisms, which could possibly include changes in protein levels and/or activity of steroidogenic gene products, may account for the effects of FOXO1 in steroid production in hLGCs.

### Antiapoptotic effects of miR-96 are conserved in bovine luteal cells

**Figure 5.**
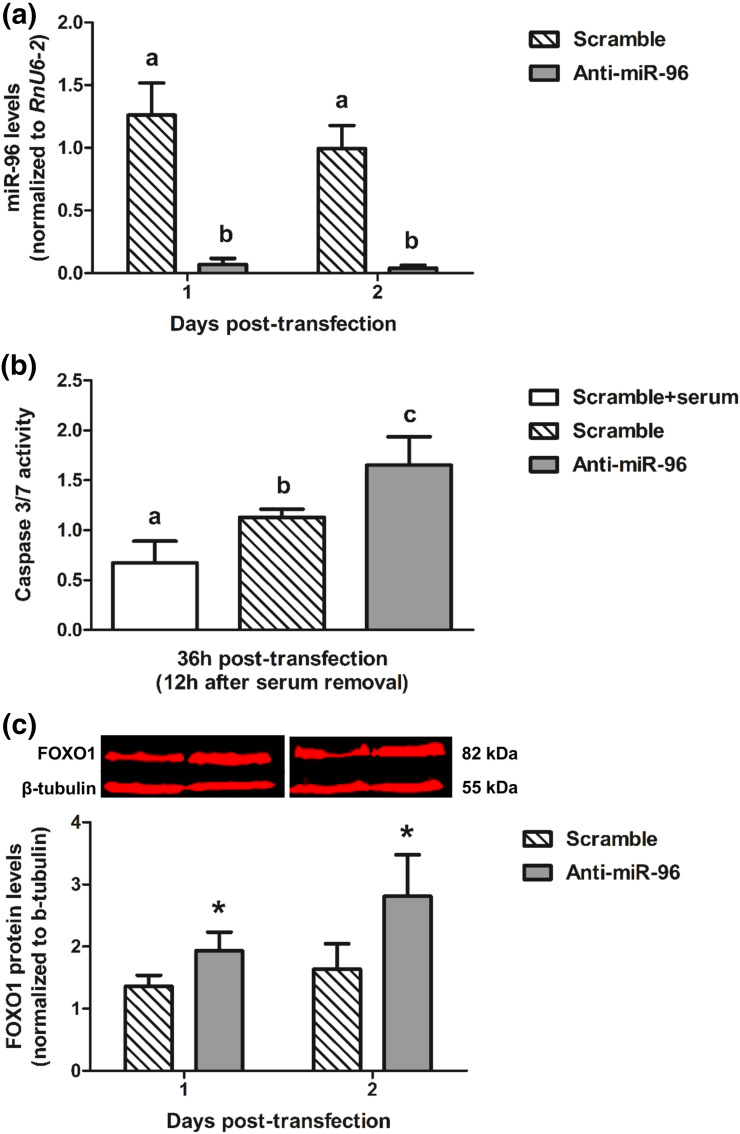
Relative levels (mean ± SE) of (a) miR-96, (b) Caspase 3/7 activity, and (c) FOXO1 protein (with representative Western blot) after transfection of bovine luteal cells with scramble oligonucleotide (50 nM, negative control) or anti-miR-96 (50 nM) at the indicated times (n = 3 experiments). Values shown for each time point were normalized in all cases to the corresponding value at the time of transfection (day 0 or hour 0). For miR-96 and Caspase 3/7, significant differences (*P* < 0.05) between means are shown by different letters (a,b,c). Mean FOXO1 values were compared between groups within each day. *P < 0.05.

Finally, to investigate the functional conservation of miR-96 during the follicle-luteal transition in other species, we established whether the observed effects of this miRNA in human ovarian cells also occurred in bovine. We collected cells from bovine CLs and cultured them in the presence or absence of anti-miR-96. Consistent with data in humans [Fig. 3(c)], inhibition of miR-96 [Fig. 5(a)] led to an increase in Caspase 3/9 activity in response to serum deprivation [Fig. 5(b)], together with an increase in FOXO1 protein levels [Fig. 5(c)]. In contrast, progesterone production by bovine cells was not affected by miR-96 inhibition (data not shown).

## Discussion

Little is known about the molecular regulation of the follicle-luteal transition in humans, to a large extent because of the limited availability of healthy ovarian tissues for study. In this regard, monovular species such as cattle can provide extremely valuable insight, particularly when compared with common rodent models with much more distinct ovarian physiology (26, 27). In this study, we demonstrate the value of this comparative approach by identifying, using the bovine model, an miRNA-mediated mechanism involved in functional regulation of the human CL.

The finding of an increase in the expression of the miR-183-96-182 and miR-212-132 clusters, as well as miR-21, during luteinization is consistent with results of previous studies in other species, including bovine (7, 9, 13, 28–30), overall suggesting conserved roles of these miRNAs during the follicle-luteal transition. However, although an effect of miR-21 in promoting cell survival during ovulation has already been demonstrated, at least in mice (16), the precise roles of the miR-183-96-182 and miR-212-132 clusters, the most upregulated miRNAs during the follicle-luteal transition, had so far not been defined during that transition.

Studies in rodents showed FOXO1 regulates different granulosa cell pathways involved in proliferation, survival, and differentiation (25, 31–33), and ovarian FOXO1 expression to rapidly terminate in response to the ovulatory gonadotropin surge (34). Moreover, FOXO1 was experimentally validated as an miR-96 target in humans (35, 36) and cattle (28). Indeed, the latter study, by Gebremedhn *et al.* (28), was the only one so far, to our knowledge, to show targeting of FOXO1 by the miR-183-96-182 cluster specifically in granulosa cells, which reportedly enhanced cell proliferation. In contrast, we found no evidence of an effect of miR-96 or miR-132 on proliferation of luteal cells (data not shown). Cell cycle arrest (rather than proliferation) associated with activation of survival pathways is a hallmark of luteinization (1), consistent with the antiapoptotic effects of miR-96 in both human and bovine cells in our study. Taken together, our results and those of Gebremedhn *et al.* (28) indicate that the effects of the miR-183-96-182 cluster may depend on the stage of follicle/luteal development. Most importantly, we identified miR-96 as a mediator of a key effect of the ovulatory luteinizing hormone surge, promotion of luteal cell survival, through targeting of FOXO1.

Granulosa-derived cells are the main source of luteal progesterone. The observed decrease, albeit short lasting, in progesterone production by hLGCs in response to anti-miR-96 indicates a stimulatory effect of miR-96 on this crucial function. In contrast, a previous study reported an inhibitory effect of miR-96 on progesterone production by human nondifferentiated granulosa cells, suggesting the effects of this miRNA may be developmental-stage specific (12). Moreover, we show the inhibitory effects of miR-96 are mediated through downregulation of FOXO1. Studies in rodent granulosa cells (25) provided evidence that FOXO1 may target several genes along the cholesterol/steroid synthesis pathway (including *hmgcs1, nr5a1, star*, *cyp11a1, cyp19a1*, and *cyp271a1*) acting to prevent a premature increase in follicular steroid production before luteinization. Our results implicate miR-96 in modulating the effect of FOXO1 on steroidogenesis in humans; however, because we did not detect significant changes in the transcripts for these cholesterol/steroid-producing genes, further investigation of the downstream mechanisms involved is warranted. In contrast to hLGCs, an effect of miR-96 inhibition on progesterone production by bovine luteal cells was not observed. Compared with human cells, bovine cells were collected from relatively mature CLs; thus, our finding suggests the stimulatory effects of miR-96 may occur only during the initial stages of luteinization. Alternatively, these results may be explained by the heterogeneous bovine luteal cell preparations used in our study, which contained not only granulosa-derived but also other luteal cell types, or may reflect intrinsic differences in regulation of luteal progesterone production between humans and cattle (*e.g.,* the distinct dependence of human granulosa lutein cells on luteinizing hormone) (1).

Because of its relative high abundance in luteal cells, allowing robust downregulation using LNAs, we focused our analyses on miR-96. However, it should be noted that all miR-183-96-182 cluster miRNAs have similar seed sequences, and there is evidence they coordinately regulate some target genes (28, 35, 37). Thus, all three miRNAs are expected to contribute to luteal regulation *in vivo*. Further complexity in predicting physiological effects of these miRNAs is provided by the fact that they presumably act in coordination with other miRNAs [*e.g.*, the antiapoptotic miR-21 (16)], thus ensuring robust follicular differentiation.

Finally, in contrast to anti-miR-96, miR-132 inhibition in our study had only small, nonsignificant effects on hLGCs, particularly in relation to cell survival. In addition, it did not enhance the effects of miR-96 alone. Yet, albeit to a slightly lower extent than anti-miR-96, miR-132 inhibition did induce an increase in FOXO1 protein, which agrees with this being an experimentally validated target of miR-132 (38, 39). Although we do not have an explanation for the relatively minor cell responses to miR-132 compared with miR-96 in the face of similar inhibitory effects on FOXO1, this may reflect differences in specific effector mechanisms elicited by the two miRNAs—a possibility that should be investigated in the future. Moreover, in line with our results, inhibition of miR-132 and miR-212 in a previous study (9) had no obvious effects on steroid production by mouse granulosa cells, overall suggesting the miR-212-132 cluster may, instead, be involved in other aspects of the follicle-luteal transition.

In summary, using a cross-species approach, we identified miR-96 as a novel regulator of the follicle-luteal transition, through FOXO1-mediated promotion of luteal cell survival and progesterone production. Reported wider roles for FOXO1 in the ovary suggest that miR-96 and, indeed, the miR-183-96-182 cluster, likely has broader effects during the follicle-luteal transition. Such effects may be important not only in ensuring a normal luteal phase but also in regulating luteal rescue and the establishment of pregnancy, a potential key link for investigation in future studies.
