# Supplementary material for: The Adequate Corpus Luteum: miR-96 Promotes Luteal Cell Survival and Progesterone Production
Source: J Clin Endocrinol Metab. 2017 Mar 20;102(7):2188–98. doi: 10.1210/jc.2017-00259 (PMC5505194; doi:10.1210/jc.2017-00259)
Supplement: Supplementary file 1 [file jc.2017-00259.st1.docx]

**Table S1. Primer sequences used in mRNA analyses**

| **Gene** | **Sequence (5’-3’) sense / antisense** |
| --- | --- |
| Bovine |  |
| *18S* | GCTGGCACCAGACTTG / GGGGAATCAGGGTTCG |
| *CYP19A1* | CGCAAAGCCTTAGAGGATGA / ACCATGGTGATGTACTTTCC |
| *CDKN1A* | GGAGACCGTGGTTGGGAGA / CTCAGACATGGCACCTGTGG |
| *HBEGF* | GGAGGAGCGTGGGAAAAGAA / TGGCACCTCTCTCCGTGATA |
| *MMP9* | CCATTAGCACGCACGACATC / GAAGGTCACGTAGCCCACAT |
| *HSD3B1* | GCGTTTCTCAGTGCTCAGATTT / TCAGCTTGATCTTGCTCTGGA |
| *ADCY6* | CTGGGGCTCGTTTATCTGGT / TGTATTTGAGTGCCACCCTCC |
| *FOXO1* | AGTGGATGGTCAAGAGCGTG / GAGCATCCACCAGGAGCTTT |
| *LHCGR* | GGACTCTAGCCCGTAGG / ACACATAACCACCATACCAAG |
| Human |  |
| *GAPDH* | GTTCGACAGTCAGCCGCATC / TCAAGGGGTCATTGATGGCA |
| *NR5A1* | GTGGTGTGAGGGGGTTTCTG / TACGAATAGTCCATGCCCGC |
| *HMGCS1* | TTGGCGGCTATAAAGCTGGTG / GCATGGTGAAAGAGCTGTGTG |
| *STAR* | ACTTGCAGGCTTCGACTAGG / CTCTTTGTGATAGGCTGCACTGT |
| *CYP11A1* | CTCCAGAAATGGTACAGGTTTAGCC / CAGGTACAGTCACAGCTGTGG |
| *CYP19A1* | TGCGAGTCTGGATCTCTGGA / CCCAAGTTTGCTGCCGAATC |
| *CYP27A1* | GATGCAGCTACTCCTCGCAA / CTCTTCAACTCCCCCGTCTC |
|  |  |
